# Natural and Designed Cyclic Peptides as Potential Antiviral Drugs to Combat Future Coronavirus Outbreaks

**DOI:** 10.3390/molecules30081651

**Published:** 2025-04-08

**Authors:** Hilarie Uwamahoro, Willard E. Collier, Toufic O. Nashar, Jesse M. Jaynes, Desmond G. Mortley, Cheryl G. Davis, Getrude G. Kanyairita, Eslam F. Abdelazim, Jean Francois Regis Igiramaboko, Concorde Habineza, Devotha Tumushimiyimana, Umuraza Noella Rutayisire, Yasmin A. Davis, Kamora L. Renard

**Affiliations:** 1 Department of Chemistry, College of Arts & Sciences, Tuskegee University, Tuskegee, AL 36088, USA; huwamahoro5872@tuskegee.edu (H.U.); jjaynes@tuskegee.edu (J.M.J.); gkanyairita5762@tuskegee.edu (G.G.K.); eabdelazim6255@tuskegee.edu (E.F.A.); 2 Department of Pathobiology, College of Veterinary Medicine, Tuskegee University, Tuskegee, AL 36088, USA; tnashar@tuskegee.edu; 3 Department of Agricultural and Environmental Sciences, College of Agriculture, Environment & Nutrition Sciences, Tuskegee University, Tuskegee, AL 36088, USA; dmortley@tuskegee.edu; 4 Department of Biology, College of Arts & Sciences, Tuskegee University, Tuskegee, AL 36088, USA; cdavis3@tuskegee.edu (C.G.D.); ydavis1256@tuskegee.edu (Y.A.D.); 5 Department of Health Sciences, Registered Nursing Program, Trenholm State Community College, Montgomery, AL 36108, USA; a02495987@alabama.edu; 6 Computational Data Science & Engineering, College of Engineering, North Carolina A&T State University, Greensboro, NC 27411, USA; chabineza@aggies.ncat.edu; 7 Department of Human Ecology, College of Agriculture, Science and Technology, Delaware State University, Dover, DE 19901, USA; dtumushimiyimana20@students.desu.edu; 8 Department of Natural Resources and Environmental Sciences, College of Agricultural, Life and Natural Sciences, Normal, AL 35811, USA; umurazanoella.ru@bulldogs.aamu.edu; 9 Department of Health Science, School of Nursing & Allied Health, Tuskegee University, Tuskegee, AL 36088, USA; krenard3984@tuskegee.edu

**Keywords:** RNA viruses, SARS-CoV-2, broad-spectrum antivirals, phytochemistry, molecular modeling, cyclic peptides

## Abstract

The COVID-19 pandemic has underscored the need for effective and affordable antiviral drugs. Anthropogenic activities have increased interactions among humans, animals, and wildlife, contributing to the emergence of new and re-emerging viral diseases. RNA viruses pose significant challenges due to their rapid mutation rates, high transmissibility, and ability to adapt to host immune responses and antiviral treatments. The World Health Organization has identified several diseases (COVID-19, Ebola, Marburg, Zika, and others), all caused by RNA viruses, designated as being of priority concern as potential causes of future pandemics. Despite advances in antiviral treatments, many viruses lack specific therapeutic options, and more importantly, there is a paucity of broad-spectrum antiviral drugs. Additionally, the high costs of current treatments such as Remdesivir and Paxlovid highlight the need for more affordable antiviral drugs. Cyclic peptides from natural sources or designed through molecular modeling have shown promise as antiviral drugs with stability, low toxicity, high target specificity, and low antiviral resistance properties. This review emphasizes the urgent need to develop specific and broad-spectrum antiviral drugs and highlights cyclic peptides as a sustainable solution to combat future pandemics. Further research into these compounds could provide a new weapon to combat RNA viruses and address the gaps in current antiviral drug development.

## 1. Introduction

Globalization, trade, and other anthropogenic activities increase contact among humans, animals, and other wildlife populations [1]. These interactions contribute to new and reemerging viral disease outbreaks that pose great public health challenges [1,2,3]. Among the potential pathogens that cause interspecies diseases, RNA viruses are of special concern [2,4]. RNA viruses have received much attention due to significant differences compared with DNA viruses in terms of their evolution, rapid transmission, mutation, and life-threatening nature [5,6]. RNA viruses pose a greater threat than DNA viruses because of their high mutation rate and rapid adaptation to new environments, including the host immune response and antiviral drugs [7]. This is due to the error-prone nature of RNA-dependent polymerases, which lack the ability to proofread misincorporated nucleotides during RNA-dependent RNA replication. Consequently, this leads to the formation of diverse variants. In contrast, DNA viruses have more robust proofreading mechanisms [7,8].

The World Health Organization (WHO) has identified several diseases caused by RNA viruses that have the potential to cause future deadly outbreaks and pandemics. These include COVID-19, Middle East respiratory syndrome (MERS), severe acute respiratory syndrome (SARS), Crimean–Congo hemorrhagic fever, Ebola virus disease, Marburg virus disease, Lassa fever, Nipah and other henipaviral diseases, Rift Valley fever, Zika, and Disease X [9]. RNA viruses responsible for causing these diseases originate from reservoir species, often mammals, and jump to humans to cause syndromes of varying forms and severity [10]. Furthermore, despite the discoveries made and the attempts to combat viral diseases, specific treatments for many viruses are lacking because of viral escape mutants [11].

Previous outbreaks and pandemics have not only overwhelmed medical and public health capacity but also created enormous strain on economic, scientific, and political systems [12]. If we are wise enough to heed their warnings, recent pandemics, especially those in the twentieth and twenty-first centuries, provide valuable insights into designing and implementing more effective strategies to combat future pandemics.

The world was unprepared for the COVID-19 pandemic and had to combat the threat through ill-conceived wellness protocols, experimental vaccines, and repurposed antiviral drugs. For example, hand sanitizers, face masks, social distancing, lockdowns, mRNA vaccines, and off-label antiviral drugs were extensively deployed to control the COVID-19/SARS-CoV-2 outbreak.

All three of these approaches to combating viral pandemics (wellness protocols, vaccines, and antiviral drugs) are crucial to ultimate success. However, all these approaches require continual research and development. While each approach has its own limitations and risks, all these approaches are vital and deserve equal funding. The COVID-19 pandemic sadly demonstrated what happens when one approach (vaccines) is favored while another approach (antiviral drugs) is neglected.

During the COVID-19 pandemic, wellness protocols included the use of personal protective equipment (PPE) such as face masks to prevent the spread of SARS-CoV-2, with different types of PPE available for public and healthcare settings [13]. However, masks had limitations including discomfort during prolonged use, breathing difficulties, and variable filtration [13]. Chemical disinfectants and sanitizers such as detergents, bleach, and alcohols were also widely used to target the outer lipid layer of the coronavirus and deactivate the viral particle [14], but their excessive use led to respiratory issues, irritation, inflammation, swelling, and problems affecting the central nervous system and gastrointestinal tract [15]. Social distancing measures, including lockdowns and stay-at-home orders, significantly impacted mental health, leading to increased anxiety, depression, stress, suicide attempts, violence, substance abuse, and domestic abuse [16]. In addition, economic hardships such as job losses and food insecurity were the direct consequences of shortsighted government responses to the pandemic [17]. Furthermore, school closures led to loss of teaching and socialization as well as loss of access to free meals and physical activity programs for children [18].

Vaccines became the preferred approach to combating the COVID-19 pandemic even though there were no approved vaccines when it started. By January 2025, the WHO had granted emergency approval for numerous vaccines to target SARS-CoV-2 variants, including mRNA vaccines (Pfizer/BioNTech Comirnaty and Moderna Spikevax) [19], non-replicating viral vector vaccines (AstraZeneca Vaxzevria, Janssen (Johnson & Johnson) Jcovden, Serum Institute of India Covishield (Oxford/AstraZeneca formulation) [19], and CanSino Convidecia [20]), inactivated vaccines (Sinopharm (Beijing) Covilo, Sinovac CoronaVac, and Bharat Biotech Covaxin) [21], and protein subunit vaccines (Novavax Nuvaxovid, Serum Institute of India COVOVAX (Novavax formulation), Corbevax [22], and SK Bioscience Co Ltd. SKYCovione [23]).

However, to adequately assess the effectiveness and safety of these new vaccines, human trials had to be conducted over significant time frames. Cases of liver damage (hepatotoxicity) were reported following vaccination against COVID-19 [24]. Another study found that adenovirus-based vaccines were associated with increased risk of blood clots (thrombosis) and low platelet levels (thrombocytopenia), while mRNA vaccines were linked to a higher risk of heart inflammation (myocarditis), with a fatality rate of 1–2 per 200 cases [25]. There is also evidence of how vaccines can trigger immune reactions leading to serious neurological harm [25]. Many countries, including the United States, Germany, France, United Kingdom, Canada, Australia, Japan, Taiwan, Singapore, and Thailand have implemented compensation programs for vaccine-related injuries and death [26]. However, such compensation cannot bring back lost lives or even reverse the harm. These previously reported findings highlight the need for continued vigilance, as similar adverse effects may occur in individuals receiving vaccines in the future.

It is even more alarming that the long-term safety of experimental vaccines is completely unknown because of the compressed time taken for their approval. It is well known that some vaccines have shown significant side effects long after the initial vaccination. For example, a study of the tetravalent dengue vaccine (CYD-TDV) in participants from 2 to 16 years of age found that vaccinated individuals were more frequently hospitalized (3%) than the control group (1.8%) [27]. This raised concerns that the vaccine increased the incidence of dengue disease in people [28]. The long-term effects of the experimental COVID-19 vaccines are completely unknown.

The third approach for fighting viral pandemics involves the use of antiviral drugs; as yet, however, there are very few specific antiviral drugs and no broad-spectrum antiviral drugs. As a 2021 editorial in *Nature* noted, “when the COVID-19 pandemic hit, the medicine cabinet was all but empty” [29]. During the pandemic, the US Food and Drug Administration (FDA) approved the use of Remdesivir to treat hospitalized patients with severe symptoms of COVID-19 [30]; unfortunately, this drug was limited and costly (>USD 3100 per patient) [31]. Some of the other drugs used to treat the disease included monoclonal antibodies such as casirivimab and imdevimab (administered together) or bamlanivimab and etesevimab (administered together)] [30]. Furthermore, in 2023, the FDA approved Paxlovid, which contains nirmatrelvir and ritonavir, for the treatment of mild to moderate COVID-19 in adults at high risk of developing severe illness [32,33]; however, the medication can cause liver problems, allergic reactions, high blood pressure, and other serious side effects [33]. The average cost of Paxlovid for 5 days of treatment is USD 1634.38 [34]. These examples highlight the need to develop more antiviral drugs to reduce treatment costs.

Many antiviral compounds have significant toxicity; for example, ribavirin used against RNA viruses and 7-deazaadenosine nucleosides (especially those with ethynyl or small hetaryl groups) showed activity against dengue and SARS-CoV-2 but exhibited significant cytotoxicity [35,36]. In addition, lethal mutagenetic agents such as Favipiravir and Molnupiravir can target RNA viruses but also cause genotoxicity and potential carcinogenic effects [36].

Antiviral drug development has long been neglected compared with vaccine development. During the COVID-19 pandemic, repurposed drugs were used extensively to fill the gap. Thus, effective and safe antiviral drugs to efficiently treat future viral threats need to be developed. Phytocompounds can contribute to the development of new antiviral drugs as they are a huge source of bioactive compounds including antivirals. Phytochemicals with antiviral activities include peptides (both linear and cyclic) and other compounds. Peptides have been found to influence membrane permeability, viral replication, and cellular functions. Therefore, they are attractive targets for antiviral research and development [37]. Specifically, cyclic peptides have attracted attention as antiviral candidates due to their stability, enzymatic hydrolysis resistance, and selectivity to receptors [38,39]. These peptides have demonstrated therapeutic potential in treating cancer and various diseases, including resistant and non-resistant bacterial, viral, and fungal infections [40]. Linear peptides are susceptible to peptidase enzyme degradation, pose off-target effects, and require routine dosing; thus, they are unstable and ineffective [41]. Furthermore, cyclic peptides offer low toxicity [40,42,43], compared with other antiviral compounds that target RNA viruses, along with high effectiveness, but they pose serious risks. The growing interest in both natural and synthetic (designed) cyclic peptide drugs can be attributed to their diverse chemical structures and biological activities, improvements in methods for their synthesis, and increased understanding of protein–protein interactions [44,45].

Overall, there is an urgent need to develop specific and broad-spectrum antiviral drugs that are effective and have low cytotoxicity in humans. This perspective review discusses the role that cyclic peptides could play as a solution to these issues, as they have low toxicity, can be easily modified to match the target, and are stable. This review explores the scientific literature to identify gaps in antiviral drug development, specifically to understand and harness the potential of natural and designed cyclic peptides to target the most recent coronavirus, SARS-CoV-2, and other coronaviruses expected to emerge in the future.

## 2. Health, Social, and Economic Consequences of the COVID-19 Pandemic

**Figure 1:**
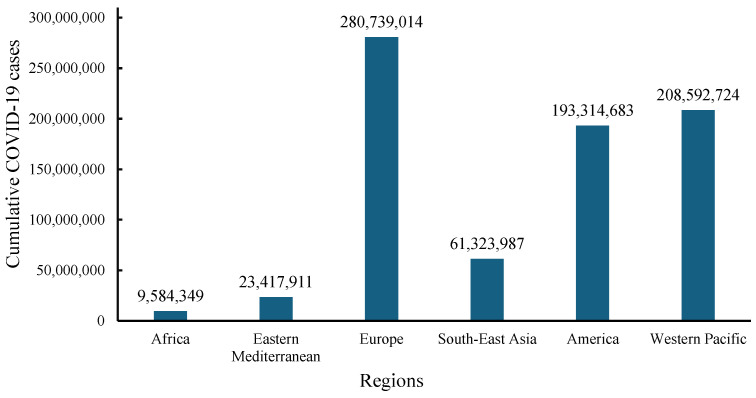
WHO data for cumulative numbers of COVID-19 cases across continents from December 2019 to 1 December 2024 [46].

**Figure 2:**
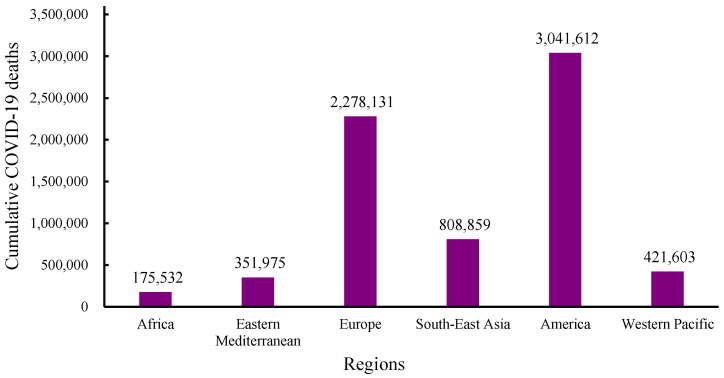
WHO data for cumulative numbers of COVID-19 deaths across continents from December 2019 to 1 December 2024 [46].

Despite efforts made in viral prevention and control, the emergence of the COVID-19 pandemic brought emotional, health, and economic shocks to the lives of many. The WHO dashboard reported that from December 2019 to 1 December 2024, total COVID-19 infections numbered 776,973,432 globally, with 7,077,725 deaths (Figure 1 and Figure 2) [46]. In December 2024, there were more than 47,230 cases globally and 521 deaths from COVID-19 within the previous 7 days. Europe had the highest cumulative infection rates, with 0.326 from 2019 to 2023 and 0.377 from 2019 to 2024 [46,47,48]. In contrast, Africa had the lowest rates, with 0.011 from 2019 to 2023 and 0.006 from 2019 to 2024 [46,47,49]. Furthermore, high- and middle-income countries experienced higher infection rates compared with low-income countries [46,47].

Table 1 shows that vaccination rates decreased with increases in dosage and/or numbers of boosters, and this trend applied across all continents. Some of the reasons may have been psychological hesitancy due to the population’s lack of confidence in vaccines, perceptions of government measures, and perceptions of the information provided, as well as the lack of convincing information [50,51]. Another interesting observation is that while Africa had the lowest vaccination rate, it also had the lowest death rate.

Africa’s low death rate while having the lowest vaccination rate might be attributable to several factors but one interesting possibility could be their extensive use of traditional medicine. Africans have a long history of using traditional plants to treat illnesses, even COVID-19, including illnesses known to be caused by viruses [52]. Research has demonstrated that plant extracts can be effective against coronaviruses, suggesting their potential as anti-SARS-CoV-2 agents [53]. Medicinal plants such as *Bryophyllum pinnatum*, *Aframomum melegueta*, *Garcinia kola*, *Sphenocentrum jollyanum*, *Adansonia digitata*, *Sutherlandia frutescens*, *Hibiscus sabdariffa*, *Moringa oleifera*, and *Nigella sativa* have shown combinations of antiviral, immunomodulatory, anti-inflammatory, and anti-COVID-19 symptom activity [52]. Methanol extracts from *Adansonia digitata* L. (Baobab) inhibited pro-inflammatory iNOS, which is an enzyme that promotes inflammation and causes the degradation of IκBα, a protein that helps in controlling inflammation. This extract also restricted the movement of NF-κB from the cytosol to the nucleus when murine macrophage RAW264.7 cells were induced with LPS (lipopolysaccharide), thereby reducing the inflammatory response in those cells [54]. Natural antiviral compounds from medicinal plants include ribosome-inactivating proteins (RIPs), terpenoids (glycyrrhizin and other specific terpenoids and lignoids), flavonoids (myricetin, scutellarein, quercetin, herbacetin, rhoifolin, pectolinarin, epigallocatechin gallate, gallocatechin gallate), phenolic compounds, etc. [11,55]. Another factor might have been that during the outbreak, several African governments acted swiftly to mitigate the spread of the virus before their healthcare systems were overwhelmed. Lockdowns, border closures, and curfews were implemented early, limiting the transmission of the virus [56]. Rwanda, Uganda, and Senegal enforced strict containment measures, including widespread mask mandates and contact tracing [56]. Also, Africa’s warmer climate may have contributed to the continent’s relatively low COVID-19 death rate. Studies in various African countries have indicated that abundant sunlight, high temperatures, higher relative humidity, and low wind speed may reduce the survivability of respiratory viruses, including SARS-CoV-2 [57].

In the United States, the advent of the COVID-19 pandemic highlighted health disparities that had been observed in earlier influenza outbreaks [58,59]. Shockingly, in the throes of the pandemic, approximately 98 out of every 100,000 African Americans died of COVID-19, exhibiting a mortality rate one-third higher than that of Latinos (65 per 100,000) and more than double the rates for both Whites (47 per 100,000) and Asians (40 per 100,000) [60]. The disproportionate representation of African Americans in confirmed COVID-19 cases and fatalities highlights the pandemic’s role in intensifying existing disparities related to race, socioeconomic status, and healthcare accessibility [60,61]. In addition, preexisting conditions such as diabetes, obesity, cardiovascular diseases, and kidney disease were significant risk factors for COVID-19 [62]. Non-Hispanic black and Hispanic/Latino adults experienced high rates of hospitalization and mortality due to COVID-19, as well as increased risk of non-COVID-19-related mortality through causes such as cardiovascular disease [63]. Throughout the COVID-19 pandemic, the non-Hispanic Black population, particularly those of advanced age, experienced challenges in securing appointments and faced affordability issues associated with obtaining medication [64]. After US Food and Drug Administration (FDA) approval, Remdesivir was the recommended treatment for patients with severe COVID-19 during the pandemic; however, it was in short supply and costly (>USD 3100 per patient) [30,31]. In the face of infection, African Americans exhibited double the hospitalization rate compared with their White counterparts [65]. Other drugs used to treat COVID-19 included monoclonal antibodies such as casirivimab and imdevimab (administered together) or bamlanivimab and etesevimab (administered together) [30], all of which were costly and in short supply. All these factors placed a disproportionate burden on African Americans. Similarly, African Americans were vaccinated at significantly lower rates compared with other groups [66]. As of 30 November 2021, race and ethnicity data had been reported for 70% of individuals who received at least one dose of vaccine. Within this group, 58% identified as White, 19% as Hispanic, 10% as African American, 6% as Asian, and 6% as multiracial, while the remaining individuals were American Indian, Alaskan Native, or Native Hawaiian or other Pacific Islanders [67]. This highlights the need for strategies that resonate with African American communities. The emergence of SARS-CoV-2 and subsequent variants has refocused attention on existing antiviral medications, especially broad-spectrum antivirals that are effective and affordable [68]. Unfortunately, there are currently only 100 approved antiviral drugs that are limited to treating only 10 out of over 220 known viruses that affect humans [69,70]. COVID-19 has underscored the urgent demand for swiftly deployable compounds to treat newly emerging or re-emerging viral diseases, particularly in situations where vaccine development is in progress [70]. Numerous antiviral agents have seen limited use in clinical treatment due to issues of ineffectiveness and resistance [71]. Minority groups, including African Americans, embrace the use of medicinal plants, being drawn to their innate therapeutic qualities and maintaining a steadfast belief in their safety [72]. Natural products characterized by potentially lower toxicity and the ability to target multiple aspects offer a promising source of compounds for effectively combating viral infections in the future [71].

## 3. Coronaviruses

Coronaviruses comprise a large family of viruses, some of which can infect humans and cause mild to severe illness, typically in the lower and upper respiratory tract [73,74]. They were first characterized in the 1960s; as of January 2025, seven coronaviruses are known to infect humans. These viruses are classified into two genera: *Alphacoronavirus* [human coronaviruses (HCoV-229E and HCoV-NL63)] and *Betacoronavirus* [human coronavirus OC43 (HCoV-OC43), human coronavirus HKU1 (HCoV-HKU1), severe acute respiratory syndrome coronavirus (SARS-CoV), Middle East respiratory syndrome coronavirus (MERS-CoV), and severe acute respiratory syndrome coronavirus 2 (SARS-CoV-2)] [73]. Coronaviruses have the largest genome (approximately 27–33 kb) among RNA viruses [75]. In the past two decades, there have been three deadly coronavirus outbreaks, all originating from bats. The first outbreak in November 2002 was SARS-CoV; its first case occurred in Foshan, China. The second was MERS-CoV in 2012, the first case of which was found in Saudi Arabia [76]. MERS was the second deadly coronavirus to have occurred in the 21st century [76]. The third was the novel coronavirus SARS-CoV-2 in 2019, which originated from Wuhan city located in Hubei Province, China.

SARS-CoV, MERS-CoV, and SARS-CoV-2 share several similarities such as originating from bats and belonging to the same *Betacoronavirus* genus in the family *Coronaviridae*, subfamily *Coronavirinae*, order Nidovirales [76]. This indicates that they possess similar genetic traits, including the capability to infect humans, as well as having a positive-sense, single-stranded RNA genome [76,77]. These viruses spread via respiratory droplets from coughing, sneezing, or talking, or through contact with contaminated surfaces. A key distinction is that SARS-CoV and MERS-CoV do not have variants, whereas SARS-CoV-2 has multiple variants including Alpha, Beta, Delta, Omicron, and others. Furthermore, SARS-CoV and SARS-CoV-2 use metallocarboxylpeptidase angiotensin receptor or angiotensin-converting enzyme 2 (ACE2) as the receptor for cell entry, whereas MERS-CoV uses the dipeptidyl peptidase 4 (DPP4) receptor [78].

### 3.1. SARS-CoV

SARS-CoV affected people worldwide, with a significant number of cases reported in Toronto, Canada. From November 2002 to July 2003, there were 8437 cases of SARS-CoV and 813 deaths. Since 2003, no further cases of the virus have been reported. Originating from bats, with civets acting as the intermediate host, the virus spread through respiratory droplets and close contact. The incubation period ranged from 2 to 10 days [78]. Symptoms of SARS-CoV included fever, cough, and shortness of breath. The fatality risk was approximately 10% [79].

### 3.2. MERS-CoV

MERS-CoV also originated from bats and was transmitted from infected dromedary camels to humans. The virus spread via direct and indirect contact with animals. There have been 2496 known cases since 2012 in 27 countries, with approximately 868 (34.77%) of cases resulting in fatalities [80]. The incubation period for MERS-CoV ranges from 2 to 10 days [78]. Symptoms of MERS-CoV infection include fever, chills, generalized myalgia, cough, shortness of breath, nausea, vomiting, and diarrhea [79].

### 3.3. SARS-CoV-2

Early cases of COVID-19 were associated with a seafood market in Wuhan/China where wild animals were also sold, which suggested that the disease was transmitted through a zoonotic spillover. Research conducted by the WHO from 14 January through 10 February 2021, indicated that the virus may have been transmitted from bats to pangolins and then to humans [81]. Another study reported civets as an intermediate host [76]. Research also revealed the high susceptibility of minks and cats to SARS-CoV-2, suggesting that more animals could have been the potential reservoir [81]. However, other researchers have conducted studies to assess the possibility of the COVID-19 pandemic being linked to a lab leak or bioweapon conspiracy theories [82,83,84]. Symptoms of SARS-CoV-2 include fever, chills, cough, difficulty breathing, sore throat, congestion or runny nose, loss of taste and smell, fatigue, muscle aches, headache, nausea, vomiting, and diarrhea [85]. The incubation period for SARS-CoV-2 ranges from 4 to 7 days [78]. As of 1 December 2024, the global fatality rate for COVID-19 was approximately 0.91% [46].

## 4. Interaction Between SARS-CoV-2 and Host Cells

One of the most important factors required in order to prevent anticipated infection by SARS-CoV-2 is a clear understanding of the molecular mechanism that lies behind its entry into the host cell. The first step is understanding the interaction between the viral infectious component and its binding receptor on the host cell.

**Figure 3:**
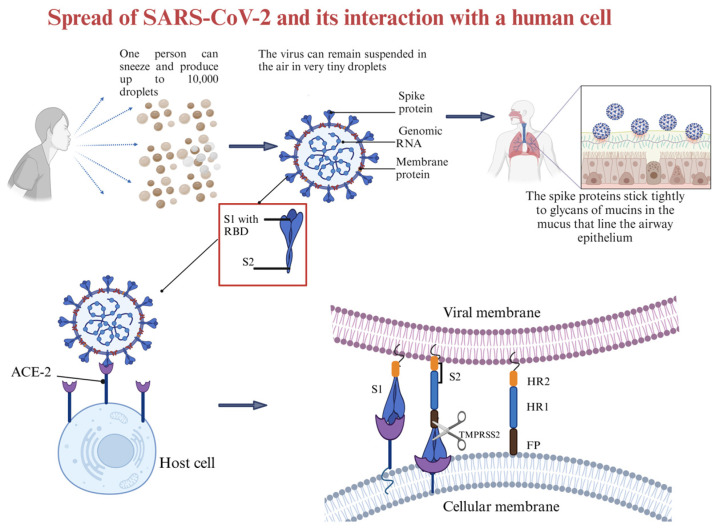
Diagram of how SARS-CoV-2 is spread and how it interacts with the host cell. Created in BioRender. Collier, W. (2025) https://BioRender.com/t31u631 (accessed 21 March 2025).

The current understanding is that coronaviruses enter host cells through one of three ways: receptor-mediated plasma membrane fusion, receptor-mediated endocytosis, or antibody-dependent viral entry [86]. In receptor-mediated plasma membrane fusion (Figure 3), SARS-CoV-2 spike protein binds with AC2 on the host cell’s membrane, triggering fusion and allowing the virus to enter and release its genetic material [87]. This was evidenced in the research conducted by Lu and Sun (2020) using assays to study the binding affinity between SARS-CoV-2 and ACE2 [88], and by Engler et al. (2023) [89] that demonstrated how SARS-CoV-2 interacts with ACE2 and EGFR (epidermal growth factor receptor). Using assays like immunoblotting analyses and siRNA-mediated knockdown, the research identified ACE2 as the primary receptor for viral entry. EGFR served as a cofactor, activating the EGFR-MAPK signaling pathway and forming a complex with ACE2, which increased SARS-CoV-2 infection in that study [89]. In receptor-mediated endocytosis, the virus binds to the host cell receptor, and the cell engulfs the virus in a vesicle, where it is internalized and releases its genetic material [90]. Ojha et al. (2024) found that without transmembrane protease serine subtype 2 (TMPRSS2), SARS-CoV-2 infects ACE2-expressing mouse embryonic fibroblasts mainly through dynamin- and actin-dependent endocytosis instead of plasma membrane fusion [91]. In antibody-dependent viral entry, antibodies specific to the virus bind to the spike protein, enhancing viral entry by facilitating fusion or promoting endocytosis [92,93]. Research conducted by Liu et al. (2021) [94] evaluated a range of anti-spike monoclonal antibodies derived from COVID-19 patients. The researchers found that certain antibodies targeting the N-terminal domain (NTD) of the spike protein promoted the open conformation of the receptor-binding domain (RBD), thereby increasing the spike protein’s binding affinity to ACE2 and enhancing SARS-CoV-2 infectivity [94].

The existence of proteins on the surface of the host cell plays an important role in maintaining the attachment of viruses to the host cell for both endocytosis and fusion [86]. The knowledge gained by studying the interaction between SARS-CoV-2 and its main receptor led to an understanding of how to block the virus from entering the host cell, which may support the development of new and promising strategies for clinical treatment and prevention [95].

The infectious component of SARS-CoV-2 is the spike protein, which consists of S1 and S2 subunits. S1 contains the RBD responsible for binding to the host cell receptor. The major cell entry receptor for SARS-CoV-2 is ACE2 [96]. ACE2 receptor expression is most prominent in the small intestine, testes, kidney, heart muscle, colon, and thyroid gland [97]. The lungs have low mRNA and protein expression levels of ACE, and blood cells do not express any [97]. S2 is made of a fusion peptide (FP), heptad repeat 1 (HR1), heptad repeat 2 (HR2), and a transmembrane domain (TM), with cytoplasmic domain fusion (CP), which is responsible for mediating viral fusion and entry [98]. The S2 subunit is highly conserved, and it is 99% homologous to human SARS-like coronavirus and the bat coronaviruses SL-CoV ZXC21 and ZC45 [98]. During viral infection, the S1 subunit is degraded and S2 is inserted into the host cell, thereby exposing three pairs of HR1 and HR2 domains that form a six-helix bundle structure that brings the cellular lipid bilayer and the viral lipid bilayer into fusion (Figure 3). TMPRSS2 is an enzyme that cleaves the spike protein into S1 and S2 subunits (Figure 3). In the respiratory tract, this enzyme is located on the cell surface, especially in the airway epithelium. After the degradation of S1 and formation of the six-helix bundle structure, the fusion process is in the pre-hairpin stage (intermediate conformation) [98]. Thus, a peptide might be designed to target HR1 and prevent conformation of the S2 subunit [98].

The mutations listed in Table 2 presented challenges relating to the discovery, use, and acceptability of vaccines. In this context, antiviral drugs, especially broad-spectrum antivirals, could have been effective for saving lives during the pandemic. However, no such broad-spectrum antiviral drug has yet been approved, which poses a big challenge in treating new and re-emerging viruses, particularly in the current context where approval is lacking for broad-spectrum antiviral drugs. Furthermore, the advent of highly pathogenic coronaviruses like SARS-CoV and MERS-CoV, along with the potential for future outbreaks caused by unknown RNA viruses, highlights the need for broad-spectrum antiviral drugs [99,100]. Traditional antiviral development has focused on targeting specific viruses, leaving us vulnerable to new or mutated strains. However, broad-spectrum antivirals offer a proactive approach to pandemic preparedness by targeting conserved mechanisms shared by various viral families [100,101]. This approach is particularly important for RNA viruses like SARS-CoV-2, which have a high mutation rate and pose a significant threat due to their rapid replication and transmissibility [101]. For example, favipiravir, an RNA-dependent RNA polymerase inhibitor, has demonstrated efficacy against a range of RNA viruses including Ebola, Lassa fever, and influenza [102,103,104]. It is important to note that both natural and synthetic cyclic peptides show potential as components of broad-spectrum antivirals [100]. These peptides can disrupt the viral envelope, inhibiting viral entry and replication. Investing in research and the development of broad-spectrum antivirals, including cyclic peptides, is essential in order to establish a robust defense against future viral threats and ensure global health security [101].

## 5. Use of Natural and Synthetic Antiviral Cyclic Peptides

**Figure 4:**
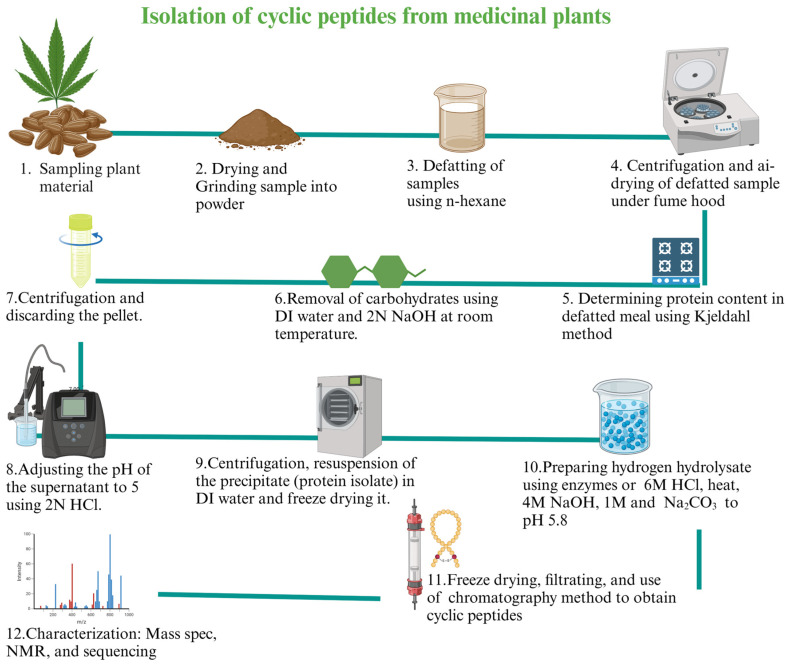
Isolation of cyclic peptides from medicinal plant seed protein isolate [109,110], N: normality, DI: deionized water. Created in BioRender. Collier, W. (2025) https://BioRender.com/z76m033 (accessed on 21 March 2025).

**Figure 5:**
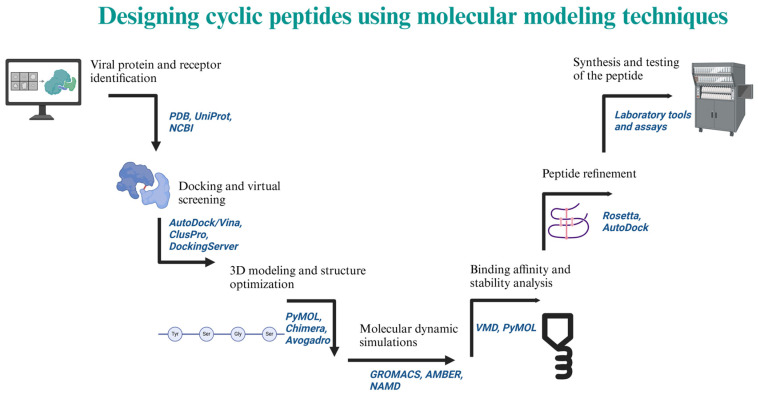
Process of designing a cyclic peptide using computational tools. Created in BioRender. Collier, W. (2025) https://BioRender.com/a47i880 (accessed on 21 March 2025).

Antiviral drugs primarily disrupt the replication of viruses by interfering with various stages of the viral life cycle, such as attachment to cells, entry into cells, uncoating, replication of either the viral DNA or RNA genome, maturation, and release of new viral particles. Phytochemical antivirals, including peptides [39], have demonstrated activity against the most recent outbreaks of coronavirus through inhibition of viral entry, replication enzymes, and mechanisms for blocking virus release [55]. These peptides can be isolated using standard protocols with some modifications (Figure 4). Designed synthetic peptide antivirals have generated significant interest due to advancements in molecular modeling techniques (Figure 5) that can predict binding interactions that enhance antiviral activity.

### 5.1. Natural Cyclic Peptides

Among natural peptides, cyclic peptides are promising drug candidates due to their stability, high target specificity, low toxicity, and resistance to hydrolysis and enzyme degradation.

**Figure 6:**
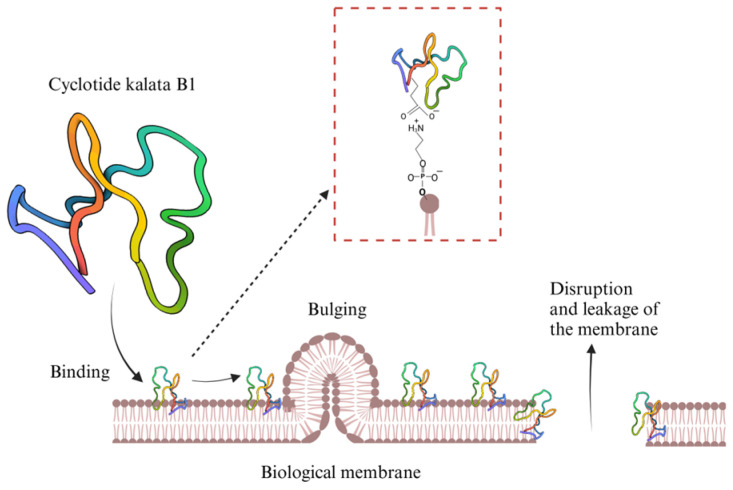
Illustration showing Cyclotides kalata B1 interacting with phosphatidylethanolamine phospholipids on a biological membrane [111,114]. Created in BioRender. Collier, W. (2025) https://BioRender.com/lj3zr5m (accessed on 21 March 2025).

Cyclotides are natural cyclic peptides, rich in disulfide bonds and with a circular backbone, that can be found in the Violaceae, Rubiaceae, Cucurbitaceae, Fabaceae, and Solanaceae plant families. They are characterized by exceptional stability [111]. Cyclotides are small proteins consisting of around 28 to 37 amino acids. Their conserved cysteine residues give them a unique knotted ring structure known as “cyclic cysteine knot (CCK)” [112]. Grover et al. (2021) examined the bioactivity of various cyclotides, 21 of which had anti-HIV properties [112]. Cyclotides have been used to target human immunodeficiency virus (HIV) and also tumor cells [111,112]. Three structures of cyclotide subfamilies, Möbius (kalata B1) [113], bracelet (cycloviolacin O2), and trypsin inhibitor (MCoTI-II), were obtained by Weidmann and Craik (2016) [111]. Cyclotide kalata B1 (CGETLKKCVGGTDCNTPGIGCTCSWPVYCTRNGLPV) specifically binds and interacts with phosphatidylethanolamine phospholipids on biological membranes, causing disruption of the membrane and leakage (Figure 6). Furthermore, the natural presence of cyclotides helps protect plants from pests and pathogens. For these reasons, cyclotides are considered promising candidates for drug applications.

In another previous study, four peptides were identified in *Acacia catechu*, each made of 15 amino acids. Each of these peptides included a similar N-terminus portion (DHVTPDIAYNP). These peptides were identified as Peptide-1 (DHVTPDIAYNPRTMY), Pepetide-2 (DHVTPDIAYNPRTYM), Peptide-3 (DHVTPDIAYNPWAFY), and Paptide-4 (DHVTPDIAYNPWAYF). These peptides demonstrated significant inhibitory activity against four serotypes of dengue virus (DENV1, DENV2, DENV3, and DENV4). The research indicated that the four identified peptides from *Acacia catechu* likely exerted their anti-DENV activity by interfering with the early stages of infection through direct interaction with the virus. The N-terminal sequence appears to be crucial for this activity, but variations in the C-terminal region can modulate the effectiveness of individual peptides. That study did not present the structures of the peptides; only their sequences were provided. No information was given with regard to whether they were linear or cyclic. The research concluded that the peptides were new and that the precise molecular mechanisms through which they blocked the initial steps of DENV infection required further investigation [115].

Meliacine is a cyclic peptide isolated from the leaves of *Melia azedarach* L.; it inhibited the multiplication of Junin virus (JV), which causes Argentine hemorrhagic fever [116]. Studies carried out in vitro showed how meliacine inhibited replication of the Junin virus by inhibiting its penetration through a blockage in the uncoating step and hindering the release of its infectious viral particles. In addition, meliacine was revealed to have the potential to inhibit the low-pH-induced fusion of infected cells, indicating its capacity to target membrane fusion events that are critical for the viral life cycle, without causing significant cytotoxicity [116]. These findings indicate the potential for meliacine to be used as an antiviral agent against the Junin virus via interference with key stages of its replication.

**Figure 7:**
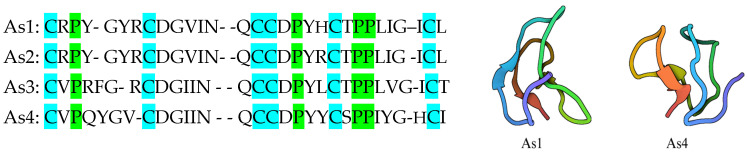
The 30-amino-acid sequences and 3D images of alstotides (As1 and As4), showing the location of the conserved cysteine knot motif and proline configuration [117,118,119].

Alstotide, also known as a cystine knot amylase inhibitor, is a cyclic peptide that was discovered in *Alstonia scholaris* and has been used to target the spike protein of SARS-CoV-2 [117]. Four alstotides were studied: As1, As2, As3, and As4. The research examined the structural characteristics of the peptides including their conserved cysteine knot motif and specific proline configurations (Figure 7), which demonstrated their stability against heat and enzymes. These peptides were found to be active against infectious bronchitis (IBV) and dengue virus, as proved by dose-dependent inhibition of plaque formation, time course assays, and other techniques. For example, the assays carried out on IBV showed the ability of As1 and As3 to inhibit IBV plaque formation according to dosage, based on reductions in viral proliferation over time [117]. Also, As1 had an antiviral effect early in the infection process, binding to the spike and membrane proteins of the IBV [117].

Singh et al. (2022) [120] evaluated a method for the virtual screening of natural peptides to identify peptides that could potentially target critical SARS-CoV-2 proteins. They used non-toxic natural antiviral peptides and comparative models to obtain 3D models of the selected peptides [120]. An existing library of natural antiviral peptides (AVPs), comprising 2060 antiviral peptides, was analyzed in that study (http://crdd.osdd.net/servers/avpdb/dd.php (accessed on 19 March 2025)). The researchers reduced the number of AVPS to 434 antiviral peptides, based on their toxicity and stability, and these were modeled using the Robetta web server (Supplementary File 1 (XLSX 32 KB): https://doi.org/10.1007/s11224-022-02113-9). The peptides were docked with viral proteins using ClusPro to study their binding affinity and to evaluate multiple target peptides. The research predicted two potential peptides that might be useful as broad-spectrum antiviral drugs. The two best AVPs identified in the study, each with high binding affinity, were sourced from conotoxin (a cyclic peptide) and DENV envelope glycoprotein (gpE) (a linear peptide) [120].

Cyclosporine is another cyclic peptide; it was originally derived from the insect-pathogenic fungus *Tolypocladium inflatum* for its antifungal activity and it was later developed into an immunosuppressant drug [121]. Cyclosporine showed activity against hepatitis C virus [122] and Rift Valley fever virus [123], and has also been reported to exert an inhibitory effect against influenza virus, rotavirus, human immunodeficiency virus, and coronavirus infections [124].

Tyrocidine A is a cyclic decapeptide isolated from *Bacillus brevis* [125]. It has been reported to exhibit strong antibiotic [126,127] and anti-malarial activity [128] while also exerting a broad-spectrum effect against fungi and viruses [127].

Daptomycin is a cyclic lipopeptide antibiotic produced by *Streptomyces roseosporus* [129] and is widely used in treating infections caused by Gram-positive bacteria. However, Cao et al. (2024) found that daptomycin interacted with the spike protein of the Omicron (B1.1.529) pseudovirus to enhance the viral cell invasion [130].

Considering the huge number of cyclic peptides that are expected to exist in nature, few natural antiviral cyclic peptides have been used against human viruses. This huge gap in the study of natural antiviral cyclic peptides represents an opportunity to find new antiviral drugs in essentially unexplored chemical space.

### 5.2. Designed and Synthetized Cyclic Peptides

Engineered cyclic peptides have been designed to bind to viral components or main proteases. Norman et al. (2021) [131] used structural design techniques to develop cyclic peptide ligands able to target the SARS-CoV-2 spike protein’s receptor-binding domain (RBD). They used mRNA display technology, peptide synthesis, and structural analysis to identify which peptides had high affinity to the receptor [131].

**Figure 8:**
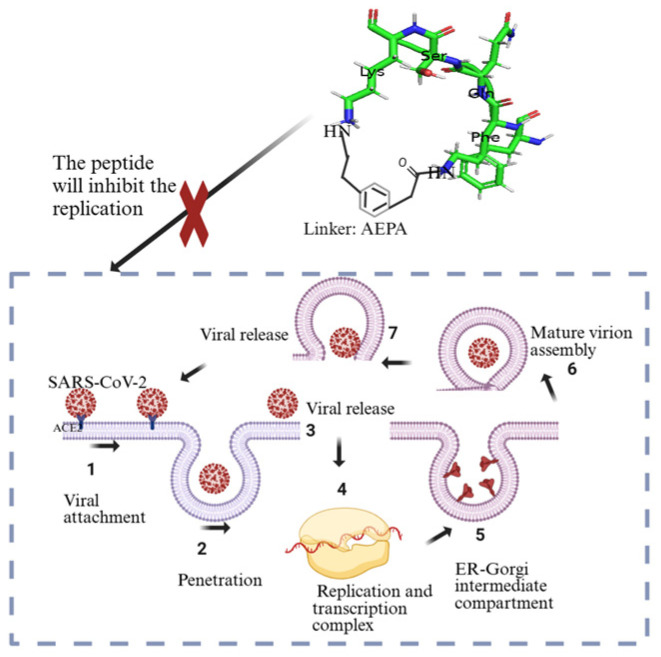
Illustration showing the role of UCI-1 pentapeptide in inhibiting viral replication [132,134]. Created in BioRender. Collier, W. (2025) https://BioRender.com/d6gpqyo (accessed on 21 March 2025).

Kreutzer et al. (2021) took an alternative approach by developing a cyclic peptide that targets the SARS-CoV-2 main protease (Mpro), aiming to inhibit viral replication rather than focusing on preventing the interaction of the spike protein with ACE2 [132]. The research designed a pentapeptide named UCI-1 (University of California, Irvine coronavirus inhibitor 1), which was a cyclic peptide consisting of four amino acids (FQSK) and one linker [4-(2-aminoethyl)phenyl]-acetic acid (AEPA) (Figure 8). This peptide was designed using part of the SARS-CoV-2 protein as a guide. Key interactions between the protease and cyclic peptide inhibitor were identified based on the structural framework of the protease, with molecular docking simulations and computation methods employed to predict peptide–protease interactions and guide the selection and refinement of peptide candidates [132]. Johansen-Leete et al. (2022) also discovered macrocyclic peptide inhibitors of SARS-CoV-2 Mpro, using RaPID (random nonstandard peptide integrated discovery) mRNA display technology on a chemically cross-linked SARS-CoV-2 main protease dimer [133].

Johnson et al. (2024) [135] investigated the effects of cyclic peptides derived from SARS-CoV-2 spike protein, which showed binding potential to human glucose-regulated protein GRP78. GRP78 is a chaperone that moves to the cell surface when a cell is under stress. A cyclic peptide was designed based on the loop structure of the SARS-CoV-2 amino acids from 480 through 488 that make up the spike protein S1 domain. The strains used to obtain the cyclic peptides were Wuhan and Omicron. Both peptides had the potential to inhibit the binding of wild-type S1 protein to GRP78. The results showed that both peptides (from the Wuhan and Omicron strains) bound to GRP78, and the Omicron cyclic peptide dissociated slowly from GRP78 [135].

**Figure 9:**
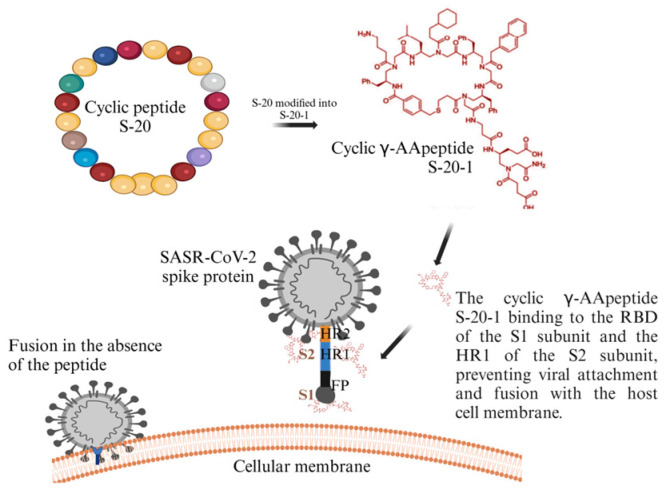
Illustration showing inhibition activity of cyclic γ-AApeptide [136]. Created in BioRender. Collier, W. (2025) https://BioRender.com/4wlued7 (accessed on 21 March 2025).

Xue et al. (2022) [136] screened a cyclic γ-AApeptide-based one-bead–two-compound (OBTC) combinatorial library (https://pmc.ncbi.nlm.nih.gov/articles/PMC5881388/ (accessed on 21 March 2025)) against SARS-CoV-2 S protein and identified a peptide called S-20, which showed strong membrane fusion and moderate safety levels. When S-20 was modified into an improved version, S-20-1 (with the addition of two negative charges), it gained the ability to inhibit infection (Figure 9) and had a safety selectivity index (SI) of over 1000. S-20-1 effectively blocked infection by authentic and pseudotyped SARS-CoV-2 as well as pseudotyped variants of concerns (VOCs), including Delta and Omicron, and other coronaviruses such as MERS-CoV, SARS-CoV, HCoV-OC43, HCoV229E, and HCoV-NL63 [136].

Jacob et al. (2024) [137] studied the validation and implementation of a multiplexed MOrPH phage display platform for discovery of macrocyclic peptides to target SARS-CoV-2 RNA, taking into consideration low micromolar affinity and high specificity. They emphasized the importance of SARS-CoV-2-1 PRFF FSS PK in the lifecycle of the virus. Their findings represent a significant step toward the development of cyclopeptide inhibitors of the -1 PRF pathway. Compounds of this type may be beneficial for future systems when used as RNA degraders [137].

Other researchers have investigated antimicrobial peptides (AMPs) as potential antiviral coatings. The peptides were obtained through synthesis. These AMPs can also be used for vaccine formulation and viral treatment. AMPs are categorized into cyclic peptides or α-helix, β-sheet, or unstructured forms, the latter including those lacking any specific spatial conformation [138,139].

## 6. Future Directions

Future research on the use of natural and designed cyclic peptides as antiviral drugs holds significant promise for combating future coronavirus outbreaks. These peptides, which can be obtained from natural sources or designed through molecular modeling, offer several advantages such as high specificity, stability, and the ability to target viral proteins with minimal toxicity to human cells. Additionally, the versatility of cyclic peptides permits the design of broad-spectrum antivirals that can target multiple variants of coronaviruses, addressing the challenges of viral mutation and resistance. Moving forward, a combination of high-throughput screening, computational modeling, and structural biology will be essential to optimize the efficacy and delivery of these peptides. Collaborative efforts between virologists, pharmacologists, and chemists will play a critical role in translating these promising antiviral candidates into safe and effective treatments for future coronavirus pandemics.

## 7. Conclusions

RNA viruses, including SARS-CoV-2 and others, have caused social, economic, and biological challenges. The pandemic caused widespread illness and increased mortality, created a major challenge for researchers, and raised concerns globally about potential future pandemics. Africa had the lowest number of cases, the fewest deaths, and the lowest percentage of vaccination coverage, while Europe had the highest cases, America had the most deaths, and the Western Pacific had the highest vaccination coverage. RNA viruses undergo rapid mutations due to a lack of proofreading mechanisms in their nucleotides, making it very challenging to provide sustainable treatments. Various approved antiviral drugs are effective; however, some of them cause high cytotoxicity. Additionally, there is a gap in the approval of broad-spectrum antivirals that can target all RNA viruses, which would help control future pandemics. Cyclic peptides from natural sources such as plants, bacteria, and fungi, as well as those designed through molecular modeling techniques, are potential candidates for sustainable and broad-spectrum antiviral drugs as they are stable, easily modified, and have low toxicity and resistance. These cyclic peptides can prevent interaction between the ACE2 receptor and the spike protein of SARS-CoV-2 by binding with either the receptor or the spike protein. It is crucial that continual antiviral drug development programs are initiated and funded on par with vaccine development, to allow researchers to keep exploring the potential of antiviral cyclic peptides. The development of antiviral cyclic peptides could be a major contribution to a long-term solution for tackling new and re-emerging viral outbreaks and pandemics around the world.

## Figures and Tables

**Table 1:** Percentages of the total population vaccinated with at least one dose, complete primary series, and at least one booster, as of 1 December 2024 [46].

Region	At Least One Dose (%)	Complete Primary Series (%)	At Least One Booster (%)
Africa	39	33	6
Eastern Mediterranean	60	52	19
Europe	69	65	37
South-East Asia	77	70	22
America	81	72	42
Western Pacific	88	86	55

**Table 2:** SARS-CoV-2 types and variants of concern, their country of origin, and key spike protein mutations in relation to the Wuhan strain.

Virus	Strains/Variants	Country of Origin	Date of Identification	Key Mutations
SARS-CoV-2	Original Wuhan strain (wild type)	China	December 2019	Wild type
	Alpha (B1.1.7)	United Kingdom	September 2020	N501Y, A570D, D614G, P681H, T716I, S982A, D1118H, and two deletions (Δ69–70 and Δ145) [105,106,107]
	Beta (B.1.351)	South Africa	May 2020	D80A, D215G, K417N, E484K, N501Y, D614G, and A701V [105,107]
	Gamma (P.1)	Brazil	November 2020	L18F, T20 N, P26S, D138Y, R190S, K417T, E484K, N501Y, D614G, H655Y, and T1027I [105,107]
	Delta (B.1.617.2)	India	October 2020	T19R, L452R, T478K, D614G, P681R, and D950N [105,107]
	Omicron (B1.1.529)	Botswana, South Africa	November 2021	A67V, T95I, Y145D, L212I, G339D, S371L, S373P, S375F, K417N, N440K, G446S, S477N, T478K, E484A, Q493R, G496S, Q498R, N501Y, Y505H, T547K, D614G, H655Y, N679K, P681H, N764K, D796Y, N856K, Q954H, N969K, L981F, three deletions (H69/V70, G142/V143/Y144, and N211), one insertion (EPE at 214) [107], and deFLiRT [108]
